# A mechanistic study on the tolerance of PAM distal end mismatch by SpCas9

**DOI:** 10.1016/j.jbc.2024.107439

**Published:** 2024-06-03

**Authors:** Dhritiman Dey, Rudra Chakravarti, Oindrila Bhattacharjee, Satyabrata Majumder, Dwaipayan Chaudhuri, Kazi Tawsif Ahmed, Dipanjan Roy, Bireswar Bhattacharya, Mansi Arya, Anupam Gautam, Rajveer Singh, Rahul Gupta, Velayutham Ravichandiran, Dhrubajyoti Chattopadhyay, Abhrajyoti Ghosh, Kalyan Giri, Syamal Roy, Dipanjan Ghosh

**Affiliations:** 1 Department of Natural Products, National Institute of Pharmaceutical Education and Research, Kolkata, West Bengal, India; 2 Plant-Microbe Interaction Division, National Institute of Plant Genome Research, Delhi, India; 3 Department of Life Sciences, Presidency University, Kolkata, India; 4 Algorithms in Bioinformatics, Institute for Bioinformatics and Medical Informatics, University of Tübingen, Tübingen, Baden-Württemberg, Germany; 5 International Max Planck Research School ‘From Molecules to Organisms’, Max Planck Institute for Biology, Tübingen, Baden-Württemberg, Germany; 6 Infectious Diseases and Immunology Division, Indian Institute of Chemical Biology, Kolkata, West Bengal, India; 7 Sister Nivedita University, Kolkata, West Bengal, India; 8 Department of Biochemistry, Bose Institute, Kolkata, West Bengal, India

**Keywords:** CRISPR-Cas9, RMSD, off-target effect, mismatch tolerance, DNA-RNA conformational stability, PAM distal end

## Abstract

The therapeutic application of CRISPR-Cas9 is limited due to its off-target activity. To have a better understanding of this off-target effect, we focused on its mismatch-prone PAM distal end. The off-target activity of SpCas9 depends directly on the nature of mismatches, which in turn results in deviation of the active site of SpCas9 due to structural instability in the RNA-DNA duplex strand. In order to test the hypothesis, we designed an array of mismatched target sites at the PAM distal end and performed *in vitro* and cell line-based experiments, which showed a strong correlation for Cas9 activity. We found that target sites having multiple mismatches in the 18th to 15th position upstream of the PAM showed no to little activity. For further mechanistic validation, Molecular Dynamics simulations were performed, which revealed that certain mismatches showed elevated root mean square deviation values that can be attributed to conformational instability within the RNA-DNA duplex. Therefore, for successful prediction of the off-target effect of SpCas9, along with complementation-derived energy, the RNA-DNA duplex stability should be taken into account.

The CRISPR-Cas9 system, utilizing the Cas9 protein from *Streptococcus pyogenes* (SpCas9), is pivotal for targeted genome editing. In type II CRISPR systems, SpCas9 is the multi-domain endonuclease that, along with a single guide RNA (sgRNA), targets specific DNA sequences with a PAM sequence, typically NGG, where N is any nucleotide. The sgRNA binds to apo-Cas9, causing a conformational change that activates SpCas9 for DNA binding. The SpCas9-sgRNA complex then stochastically searches and binds to DNA sequences complementary to the sgRNA's first 17 to 20 nucleotides at the 5′ end. This binding leads to the cleavage of the target DNA between the third and fourth nucleotide upstream of the PAM, creating a Double-Stranded Break (DSB), which can be repaired *via* non-homologous end joining (NHEJ) or homology-directed repair (HDR) (1, 2, 3, 4, 5, 6). The PAM site is crucial for SpCas9 binding initiation, while the seed sequence, located near the 3′ end of the sgRNA, is critical for subsequent SpCas9 binding, R-loop formation, and nuclease activation. The Cas9-sgRNA complex's crystal structure shows that the HNH and RuvC nuclease domains are responsible for cleaving the complementary and non-complementary DNA strands, respectively (6).

Recent studies have shown that the correct interaction of the gRNA with the targeted DNA sequence is essential and plays a crucial role in the correct functioning of the Cas9 endonuclease activity (7). Although CRISPR is a fast, efficient, and cheap genome editing technique, its off-target effects, give rise to genetic instability causing unwanted phenotypes thus limiting its application for therapeutic purposes (8, 9). There are many reasons for the off-target effects of CRISPR systems; the tolerance of mismatches in the PAM distal end is one of the most important ones. SpCas9 has been shown to tolerate mismatches in the PAM distal end (10, 11) while being much more conservative in the seed region and its adjacent areas. This tolerance is explained by the stabilization of the distorted duplex by a domain loop that penetrates this duplex (12). Even, the bridge helix arginine-rich domain and REC lobe of the Cas9 protein also hold the key for off-target activities (13).

This work aims to shed light on the effect of the number and position of mismatches in the PAM distal end of target DNA on the functional activity of SpCas9. We have also tried to give a mechanistic insight into the tolerance of various types of mismatches in the PAM distal end on the functional activity of SpCas9 through Molecular Dynamics (MD) simulation. The tolerance profile was found to vary with the nature of the mismatch that was attributed toward the stability of DNA–RNA duplex. This variation may also result due to variation in GC content between different sequences (14). In other words, the significance given to one position may not hold much value when compared across other sequences of varied GC content. Due to this, we suggest using structural instability of the RNA: DNA duplex in predicting tolerance of mismatches. We have studied the effect on SpCas9 activity, upon mismatches and deletion in the complementary PAM distal end of the gRNA probe. Furthermore, we have also studied many varieties of staggered mismatches and their activities using *in vitro* cleavage assay and cell-based reporter assay (Fig. 1, *A*–*D*). The results from cell-based assays closely mirrored those obtained from our *in vitro* experiments. We have shown a high tolerance for various mismatches which is difficult to explain based on energy or position. To gain a more comprehensive understanding of our *in vitro* and cell-based findings, we performed molecular dynamics simulations. We have chosen 5Y36 for the study as it is denoted as the cleavage-compatible conformations with the target DNA (15). Several other structures for the CRISPR-Cas9 system with RNA and DNA are deposited in the PDB but in all of that, residue 840 (mainly His840, which is the residue responsible for the nucleophilic attack to the phosphorus atom by a water molecule) of HNH domain is very distant from the scissile P atom which resides between bases 3 and 4 upstream of PAM. This phosphodiester bond (P-O) is cleaved by a one-metal-ion hydrolysis mechanism. 5Y36 is solved with two Alanine substitution mutations in the protein part at position 10 and position 840. Asp10 resides in the RuvC domain which helps in binding two Mg2+ ions along with Glu762, Asp986. Then using a two-metal-ion hydrolysis mechanism, the phosphodiester bond between bases −4 and −3 of the non-target strand is hydrolyzed by His983 or His982 (3, 5, 16). These two substitution mutations cause loss function in the CRISPR-Cas9 We computed the root mean square deviation (RMSD) of RNA–DNA double helix using all the heavy atoms. We have selected bases 1 to 19 from RNA (chain B) and bases 21 to 39 from DNA (chain C) because this segment comprises the double helix structure. Mutations have been introduced in the region spanning bases 31 to 39 of DNA chain C (Fig. 1, *E* and *F*). Molecular dynamics simulations revealed that certain mismatch mutations induced pronounced conformational instability within the RNA-DNA duplex, leading to elevated root mean square deviation (RMSD) values. Conversely, other mutations had negligible effects on the duplex stability. This contrast in stability directly translated into the observed catalytic activity of the mutants. Specifically, those mutants characterized by a highly stable RNA-DNA duplex exhibited full catalytic activity, while mutants associated with significant duplex instability displayed negligible or no catalytic activity. A subset of mutants demonstrated intermediate stability in the duplex, resulting in partial catalytic activity. This study signifies the correlation between the effects of mismatches, on SpCas9 functional activity with the stability of the DNA: RNA hybrid. This correlation will play a key role in designing sgRNA for high-precision genome engineering studies and will lead to the development of new sgRNA prediction algorithms.

**Figure 1:**
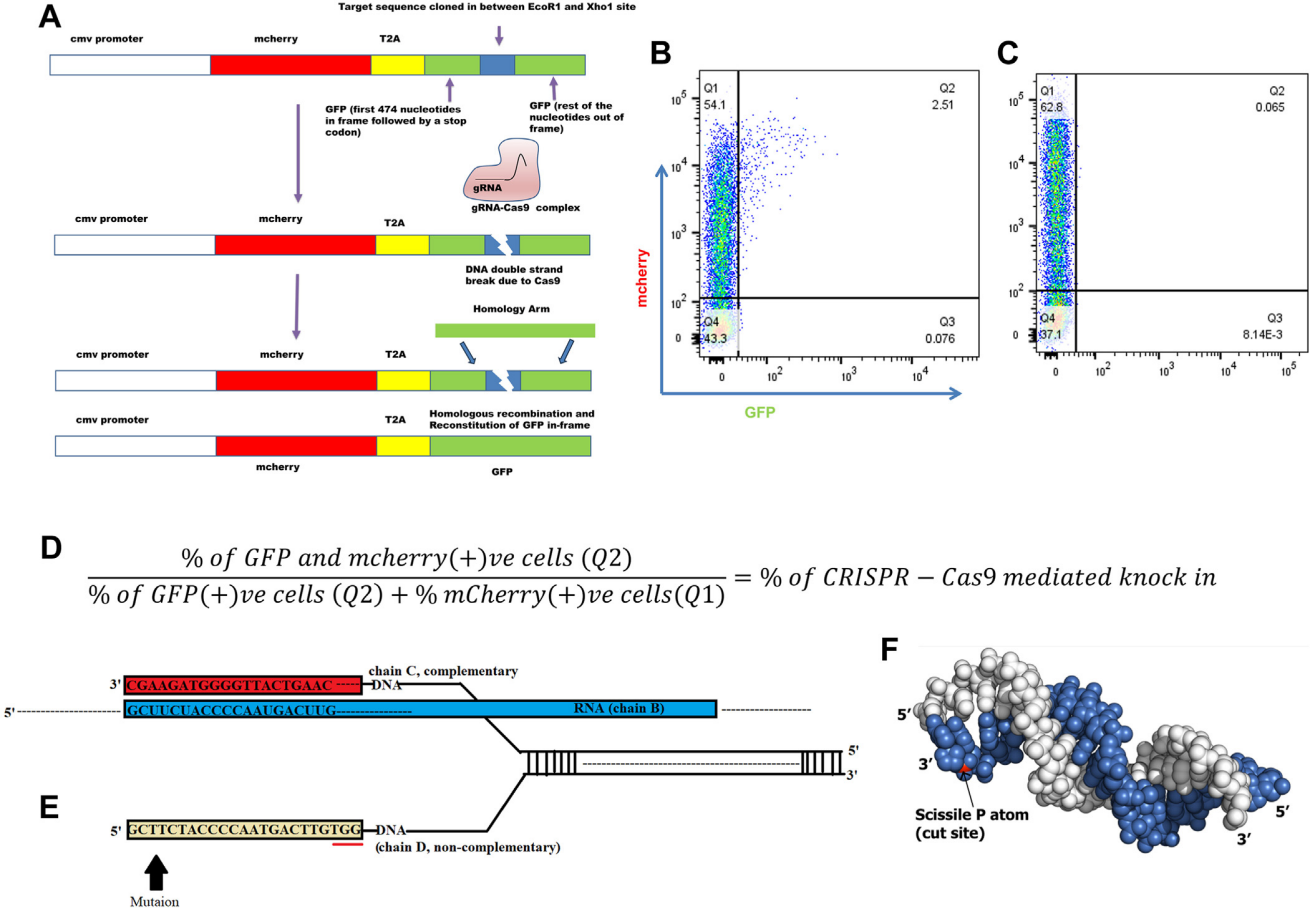
**Schematic diagram of reporter assay system and model for in silico studies.***A*, functional module of the reporter assay vector system. FACS representation of cell-based knock-in. Q1 represents mcherry positive population (transfected cells). Q2 represents dual positive population (HDR knock in population amongst transfected population). Q3 represents GFP positive population and Q4 represents non-transfected cells. *B*, it represents a species where HDR knock-in occurred (Q3 positive), and (*C*) represents a species where HDR knock-in did not occur. Each dot plot represents data from 10,000 cells. *D*, equation depicting the percentage of knock-in calculated from FACS-based experiments. *E*, sequence composition of the DNA-gRNA-Cas9 system and site for introduction of mismatch mutation. *F*, the duplex structure of DNA–RNA complex comprising RNA (base 1–19, depicted as *white spheres*) and DNA (base 21–39, depicted as *blue spheres*). The phosphorus atom at the cleavage site (scissile P) is shown in *red*.

## Results

### Cas9 activity upon variation in gRNA probe length and GC content of target site

**Figure 2:**
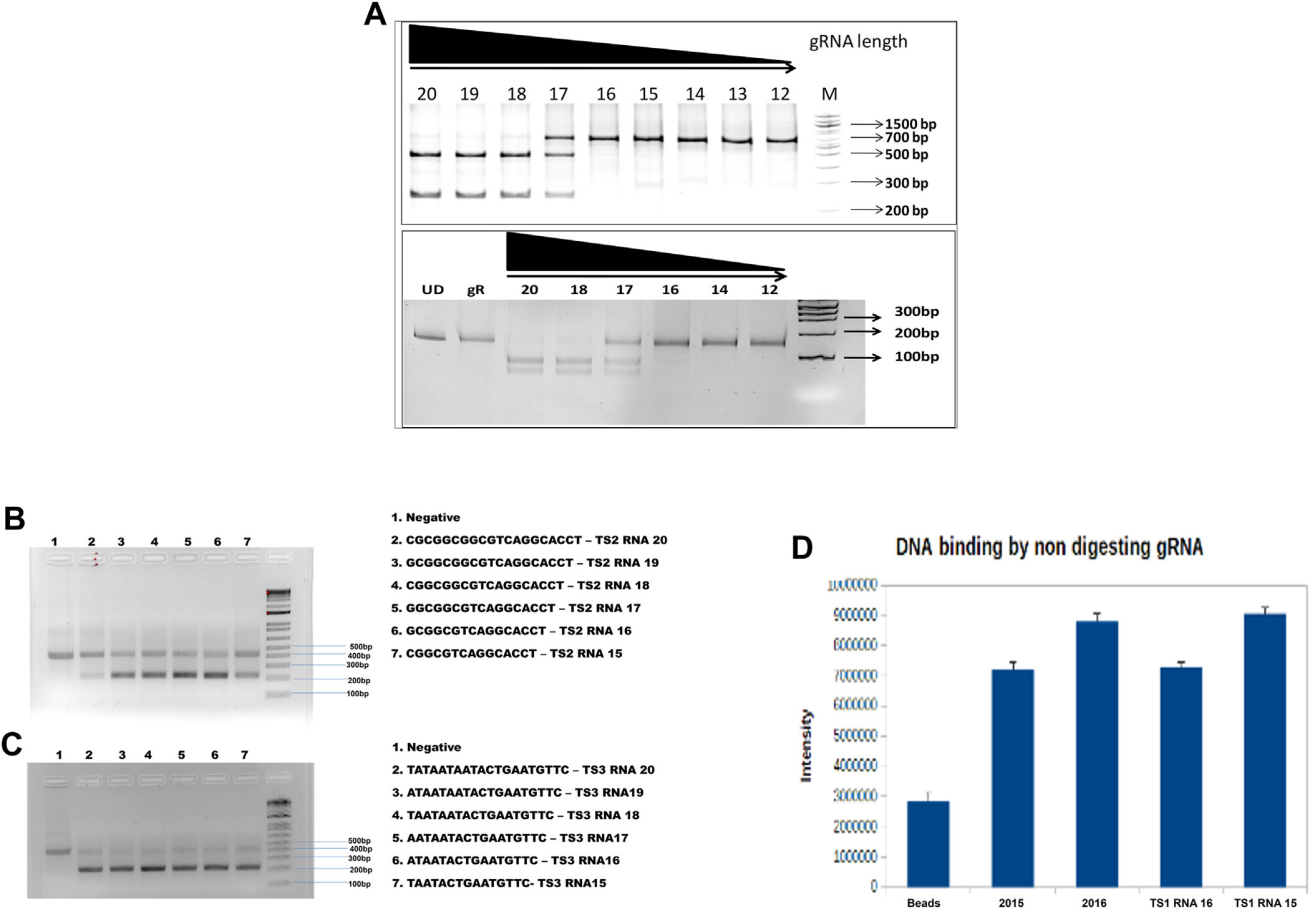
**Cas9 functionality upon using truncated gRNA probes.***A*, DNA digestion with truncated gRNA probe TS1 – DNA digestion is dependent on RNA length. 20 nt probe length to 18 nt probe length is sufficient to produce digestion. However, at 17-nucleotide length, partial digestion is produced. 16 nucleotides or less produced no digestion. 2 sets of different primers were used to obtain different amplicon sizes as validated by the markers. *B*, DNA digestion with truncated gRNA probe TS2 – DNA digestion is not dependent on RNA length. *C*, DNA digestion with truncated gRNA probe TS3 – DNA digestion is again not dependent on RNA length. *D*, pull-down of Cas9-gRNA complex using Affinity Chromatography—pull-down of Cas9-gRNA complex with higher bound DNA than control beads indicating the ability of these gRNA to bind their target DNA.

Our first aim was to study whether energy consideration holds across various sequences for Cas9 activity. We began our investigation by deleting one residue at a time from the PAM distal end and observing its effect on Cas9 functional activity. The target sequence selected had a 50% GC content target site DNA-TS1. Our results demonstrate that SpCas9 activity is sensitive to gRNA probe length modifications in TS1 (**GCUUCUACCCCAAUGACUUG)** from the PAM distal end. For getting full activity from SpCas9, up to 18 nucleotide probe length is sufficient. However, there is a severe drop in the activity when the probe length is reduced to 17 nucleotides. Furthermore, when the probe length is reduced to 16 nucleotides or less, the activity is almost abolished (Fig. 2*A*). However, it is not known whether the gRNA probe is unable to bind the target DNA or post-binding does not have sufficient energy to drive the reaction. To ascertain this, we performed a pull-down experiment with non-digesting gRNA (16 nucleotides, 15 nucleotides, 2015mm-TS1 and 2016mm-TS1). Our results show that all the gRNAs can bind the target DNA (Fig. 2*D*). This indicates that there is a cut-off complementarity required. The *in silico* energy calculation, based on complementarity with the DNA target, defines the minimum cut-off energy (ΔG) for this target as −15.6 kcal mol^−1^ (Table 1). TS1 with 16 nucleotides has ΔG of-14.2 kcal mol−1, which according to our digestion data is insufficient to drive Cas9 for even minimum activity. However, this value of minimum energy is not applicable for TS2 and TS3, with 80% and 20% GC content respectively. While in TS3, even after truncating up to 12 nucleotides activity remained. This shows that at much lower energy Cas9 was still active. However, in TS2 this was not the case. We found that Cas9 activity was inconsistent across various truncations of TS2 and TS3 (Table 2, Table 3 and 3) (Fig. 2, *B* and *C*). This is despite TS2 having 80% GC content. Thus, having sufficient scope for complementation-derived energy (Table S1, *E* and *F*). It has been reported that if the GC content of the target site is high (80%), then there is an anomalous pattern of Cas9-mediated digestion. The percentage of digestion is reduced drastically. This result is at par with the findings of Wang *et al.* in which they reported reduced efficiency of Cas9 with gRNA probes having abnormal proportions of GC content (14). With the above results, we can say that to drive the reaction forward, even to the extent of partial activity, complement length-derived energy alone cannot be the sole factor. Even with smaller probe designs, this effect will not be mitigated (17). Thus off-target predicting algorithms with only energy consideration will not suffice. This simple experiment demonstrates this and clearly shows that selections of target probes, based on energy criteria alone, may be prone to off-target activity.

**Table 1:** RNA drop

NAME	Sequence (5’>3′)	ΔG^o^(kcal mol−1)
TS1 RNA 20	GCUUCUACCCCAAUGACUUG	−17.8
TS1 RNA 19	CUUCUACCCCAAUGACUUG	−15.8
TS1 RNA 18	UUCUACCCCAAUGACUUG	−15.4
TS1 RNA 17	UCUACCCCAAUGACUUG	−15.6
TS1 RNA 16	CUACCCCAAUGACUUG	−14.2
TS1 RNA 15	UACCCCAAUGACUUG	−13.8
TS1 RNA 14	ACCCCAAUGACUUG	−13.3
TS1 RNA 13	CCCCAAUGACUUG	−11.8
TS1 RNA 12	CCCAAUGACUUG	−10.1
TS2 RNA 20	CGCGGCGGCGUCAGGCACCU	−28.6
TS2 RNA 19	GCGGCGGCGUCAGGCACCU	−27.2
TS2 RNA 18	CGGCGGCGUCAGGCACCU	−25.2
TS2 RNA 17	GGCGGCGUCAGGCACCU	−23.8
TS2 RNA 16	GCGGCGUCAGGCACCU	−21.5
TS2 RNA 15	CGGCGUCAGGCACCU	−19.5
TS3 RNA 20	UAUAAUAAUACUGAAUGUUC	−12.9
TS3 RNA 19	AUAAUAAUACUGAAUGUUC	−12.4
TS3 RNA 18	UAAUAAUACUGAAUGUUC	−12
TS3 RNA 17	AAUAAUACUGAAUGUUC	−11.5
TS3 RNA 16	AUAAUACUGAAUGUUC	−10.8
TS3 RNA 15	UAAUACUGAAUGUUC	−10.4

**Table 2:** list of Target DNA with mismatches in accordance to TS1

NAME of target	Sequence (5’>3′)
TS1	**GCTTCTACCCCAATGACTTG**
TS2	**CGCGGCGGCGTCAGGCACCT**
TS3	**TATAATAATACTGAATGTTC**
TS4	**GCTTACAACATTGTGAACGA**
TS5	**GCGTACGACATCGCAGACTG**
20mm-TS1	**ACTTCTACCCCAATGACTTG**
19mm-TS1	**GTTTCTACCCCAATGACTTG**
18mm-TS1	**GCCTCTACCCCAATGACTTG**
17mm-TS1	**GCTCCTACCCCAATGACTTG**
16mm-TS1	**GCTTTTACCCCAATGACTTG**
15mm-TS1	**GCTTCCACCCCAATGACTTG**
14mm-TS1	**GCTTCTGCCCCAATGACTTG**
13mm-TS1	**GCTTCTATCCCAATGACTTG**
2019mm-TS1	**ATTTCTACCCCAATGACTTG**
1918mm-TS1	**GTCTCTACCCCAATGACTTG**
1817mm-TS1	**GCCCCTACCCCAATGACTTG**
1716mm-TS1	**GCTCTTACCCCAATGACTTG**
1615mm-TS1	**GCTTTCACCCCAATGACTTG**
1514mm-TS1	**GCTTCCGCCCCAATGACTTG**
1413mm-TS1	**GCTTCTGTCCCAATGACTTG**
1312mm-TS1	**GCTTCTATTCCAATGACTTG**
2018mm-TS1	**ATCTCTACCCCAATGACTTG**
2017mm-TS1	**ATCCCTACCCCAATGACTTG**
2016mm-TS1	**ATCCTTACCCCAATGACTTG**
2015mm-TS1	**ATCCTCACCCCAATGACTTG**
1917mm-TS1	**GTCCCTACCCCAATGACTTG**
1816mm-TS1	**GCCCTTACCCCAATGACTTG**
1715mm-TS1	**GCTCTCACCCCAATGACTTG**
20/18mm-TS1	**ACCTCTACCCCAATGACTTG**
20/18/16mm-TS1	**ACCTTTACCCCAATGACTTG**
20/18/16/14mm-TS1	**ACCTTTGCCCCAATGACTTG**
20/18/16/14/12mm-TS1	**ACCTTTGCTCCAATGACTTG**
20/16mm-TS1	**ACTTTTACCCCAATGACTTG**
20/14mm-TS1	**ACTTCTGCCCCAATGACTTG**
18/16mm-TS1	**GCCTTTACCCCAATGACTTG**
18/14mm-TS1	**GCCTCTGCCCCAATGACTTG**
16/14mm-TS1	**GCTTTTGCCCCAATGACTTG**
20/1817mm-TS1	**ACCCCTACCCCAATGACTTG**
20/18/1615mm-TS1	**ACCTTCACCCCAATGACTTG**
18/1615mm-TS1	**GCCTTCACCCCAATGACTTG**
18/1514mm-TS1	**GCCTCCGCCCCAATGACTTG**
1716mm-TS4	**GCTCGCAACATTGTGAACGA**
18/1514mm-TS4	**GCGTATGACATTGTGAACGA**
20/1817mm-TS4	**ACGCACAACATTGTGAACGA**
20/18/1615mm-TS4	**ACGTGTAACATTGTGAACGA**
1715mm-TS4	**GCTGTGAACATTGTGAACGA**
1918mm-TS4	**GAGTACAACATTGTGAACGA**
20/16mm-TS4	**ACTTGCAACATTGTGAACGA**
1817mm-TS4	**GCGACAACATTGTGAACGA**
1514mm-TS4	**GCTTATGACATTGTGAACGA**
1716mm-TS5	**GCGATCGACATCGCAGACTG**
18/1514mm-TS5	**GCTTAACACATCGCAGACTG**
20/1817mm-TS5	**ACTAACGACATCGCAGACTG**
20/18/1615mm-TS5	**ACTTCTGACATCGCAGACTG**
1715mm-TS5	**GCGGTTGACATCGCAGACTG**
1918mm-TS5	**GATTACGACATCGCAGACTG**
20/16mm-TS5	**ACGTTCGACATCGCAGACTG**
1817mm-TS5	**GCTCACGACATCGCAGACTG**
1514mmTS5	**GCGTAATACATCGCAGACTG**

**Table 3:** sequences of gRNA probes corresponding to TS1, TS2, TS3, TS4 and TS5

Name of gRNA probe	Sequence (5’>3′)
TS1 RNA 20	**GCUUCUACCCCAAUGACUUG**
TS1 RNA 19	**CUUCUACCCCAAUGACUUG**
TS1 RNA 18	**UUCUACCCCAAUGACUUG**
TS1 RNA 17	**UCUACCCCAAUGACUUG**
TS1 RNA 16	**CUACCCCAAUGACUUG**
TS1 RNA 15	**UACCCCAAUGACUUG**
TS1 RNA 14	**ACCCCAAUGACUUG**
TS1 RNA 13	**CCCCAAUGACUUG**
TS1 RNA 12	**CCCAAUGACUUG**
TS2 RNA 20	**CGCGGCGGCGUCAGGCACCU**
TS2 RNA 19	**GCGGCGGCGUCAGGCACCU**
TS2 RNA 18	**CGGCGGCGUCAGGCACCU**
TS2 RNA 17	**GGCGGCGUCAGGCACCU**
TS2 RNA 16	**GCGGCGUCAGGCACCU**
TS2 RNA 15	**CGGCGUCAGGCACCU**
TS3 RNA 20	**UAUAAUAAUACUGAAUGUC**
TS3 RNA 19	**AUAAUAAUACUGAAUGUUC**
TS3 RNA 18	**UAAUAAUACUGAAUGUUC**
TS3 RNA 17	**AAUAAUACUGAAUGUUC**
TS3 RNA 16	**AUAAUACUGAAUGUUC**
TS3 RNA 15	**UAAUACUGAAUGUUC**
TS4 RNA 20	**GCUUACAACAUUGUGAACGA**
TS5 RNA 20	**GCGUACGACAUCGCAGACUG**

### Single-nucleotide mismatches in SpCas9-gRNA complex interaction with targeted DNA TS1

**Figure 3:**
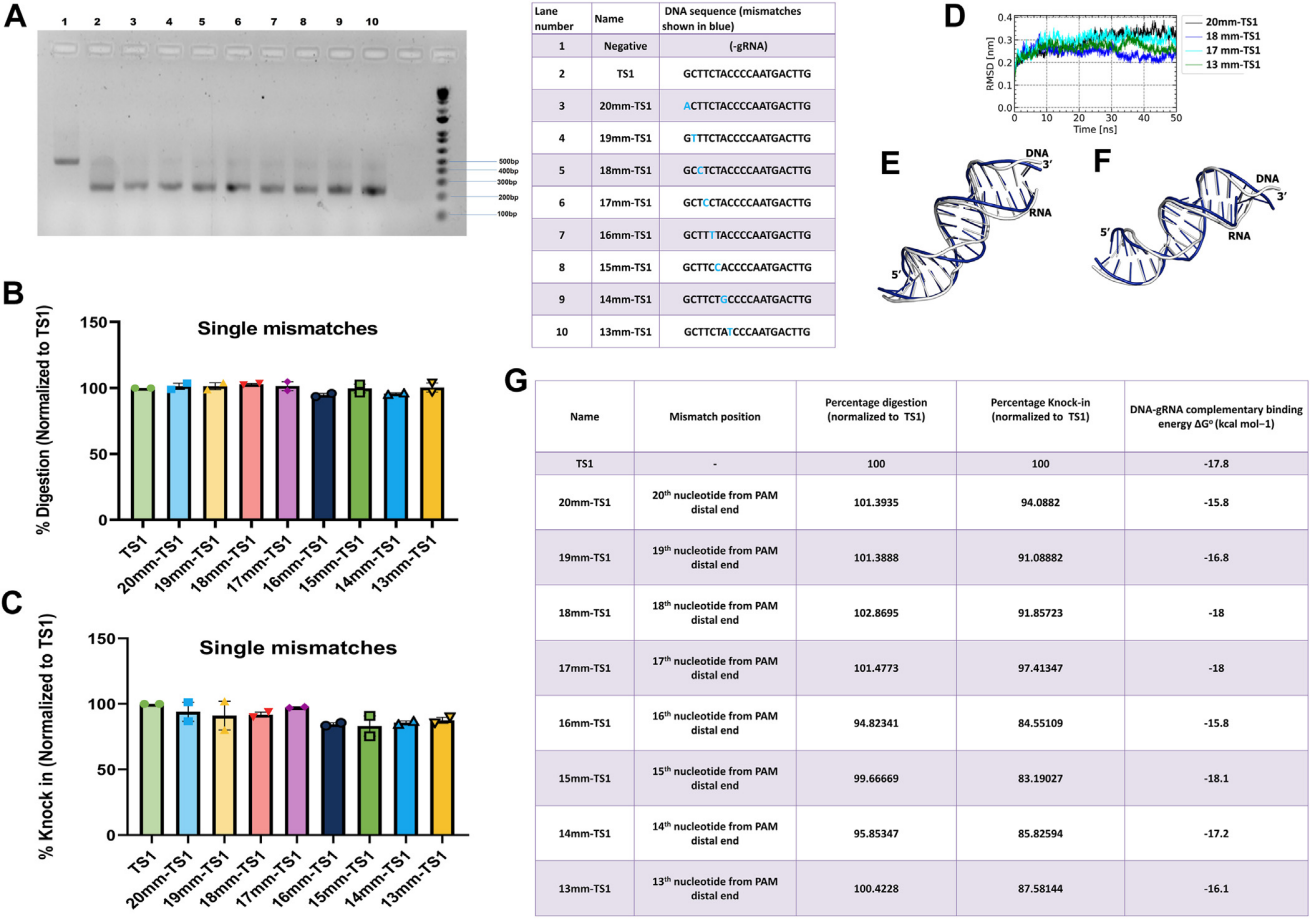
**Cas9 functional activity on encountering single mismatches across various positions.***A*, Cas9 digestion activity of TS1 and single positional mismatches in the TS1 target DNA - Digestion similar to TS1. *B*, graphical representation of the percentage digestion after 60 min. Data was normalized to TS1, (*C*) Graphical representation of the percentage of HDR knock in derived from cell-based reporter assay. Data was normalized to TS1, (*D*) RMSD trajectory of the RNA-DNA duplex from single mismatch mutants. Superimposition of initial and average conformation (computed from 40 ns–50 ns) of RNA-DNA duplex of (*E*) 18mm-TS1 (RMSD 2.4 Å) and *F*) 13mm-TS1 (RMSD 2.6 Å). *G*, tabular representation of nature of mismatch, normalized % of *in vitro* digestion, normalized %of knock in (cell-based reporter assay), and DNA-gRNA complementation derived energy.

We have observed the effects of nucleotide deletion on the activity of the Cas9: gRNA complex. With different target sites, the behavior of Cas9 was different. It is thus difficult to predict by simply emphasizing the energy derived through complementary binding. Another aspect that has been widely reported as a factor in off-target activities is the position at which the mismatch is occurring. There are reports of the position 17 to 14 being important. However other reports show that the distal end from PAM is mutation-prone. Other reports designate 13 or so from the PAM distal end as mismatch-prone (18, 19).To address this we designed many mismatched target sites derived from TS1. We now kept the length of the probe the same but sequentially mutated the TS1 site to simulate a variety of mismatches. They were divided into categories, Single mismatch, sequential mismatch, bi-sequential mismatch, and staggered mismatch. To study these mismatches—Cas9 digestion assay, cell-based knock-in in the assay, energy calculations, and finally Molecular dynamics simulation were performed. As per general expectations, single mismatches were easily tolerated (Fig. 3, *A*–*C*). The DNA: RNA binding energy was also similar to TS1 (Fig. 3*G*, Table S1*A*). Single mismatch mutations did not induce noteworthy structural deviations in the RNA-DNA duplex, as evidenced by the consistently low Root Mean Square Deviation (RMSD) values observed during the entire simulation. The high stability of the duplex conformations allowed the catalytic domain (HNH domain) to execute cleavage without encountering any obstacles, resulting in these mutants displaying full enzymatic activity (>85% digestion). We conducted structural comparisons between the initial and average conformations (extracted from the 40 ns to 50 ns timeframe) of 18mm-TS1 (Fig. 3*E*) and 13mm-TS1 (Fig. 3*F*) to visually illustrate the observed deviations. In staggered mismatch mutants, two categories emerged: highly stable duplexes (low RMSD) with high catalytic activity and considerably less stable duplexes (high RMSD) with low catalytic activity. Furthermore, no significant instability was found in the duplex upon single-nucleotide mismatches (Fig. 3*D*). Our results indicate that single nucleotide mismatches are well tolerated irrespective of their position duplex strand. Near full activity is observed at all the positions in the gRNA probe tail (Fig. 3, *A* and *B*). This shows that Cas9 can tolerate a single nucleotide mismatch, irrespective of the position of the mismatch in the PAM distal end therefore, if there is a single mismatch at the PAM distal end then it is best to not proceed as our results indicate there is a possibility of the mismatch being tolerated.

### SpCas9–gRNA complex interaction with sequentially mutated nucleotides in TS1

**Figure 4:**
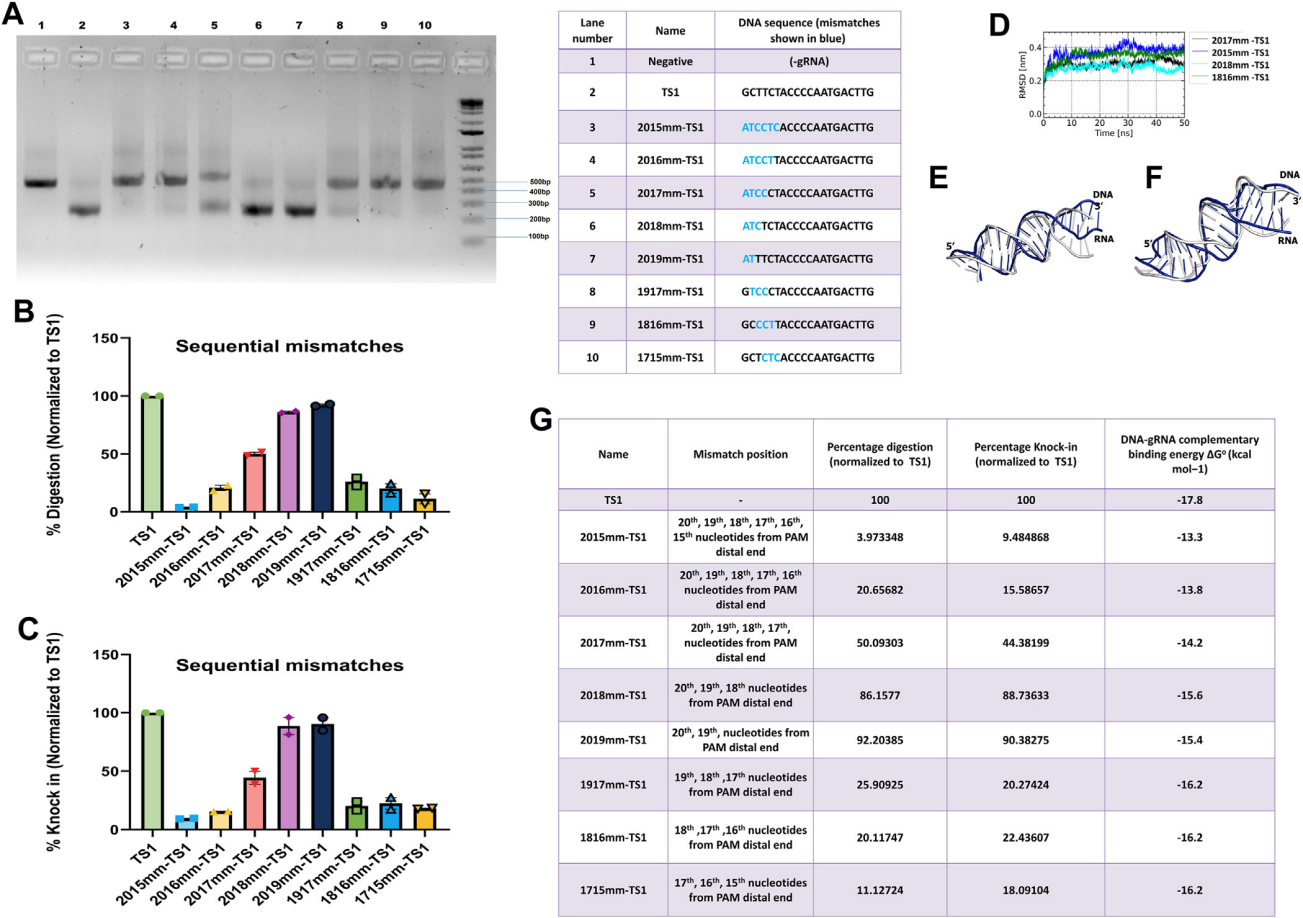
**Cas9 functional activity on encountering sequential mismatches across various positions.***A*, DNA digestion of Cas9 and sequential positional mismatches in the TS1 target DNA - Cas9 has very little activity when there are multiple sequential mutations. *B*, graphical representation of the percentage digestion after 60 min. Data was normalized to TS1. *C*, graphical representation of the percentage of HDR knock in derived from cell-based reporter assay. Data was normalized to TS1. *D*, RMSD trajectory of the RNA-DNA duplex from sequential mismatch mutants. Superimposition of initial and average conformation (computed from 40 ns–50 ns) of RNA-DNA duplex of (*E*) 2015mm-TS1 (RMSD 3.7 Å) and (*F*) 2018mm-TS1 (RMSD 2.8 Å). *G*, tabular representation of nature of mismatch, normalized % of *in vitro* digestion, normalized %of knock in (cell-based reporter assay), and DNA-gRNA complementation derived energy.

Five Sequential mutations were made from the PAM distal end and three triple sequential mutations were designed for the 19th position onwards (Table 2). This was done to gauge the effect of sequential mutation on Cas9 activity. We found that from sequential tri-nucleotide mismatch to sequential hexanucleotide mismatch there was minimum DNA cleavage activity observed. At 2017mm-TS1 (17 nucleotide complementarity) partial activity (∼50% digestion) is found while at 2016mm-TS1 (16 nucleotide complementarity) there is almost no activity (∼20% digestion) (Fig. 4, *A*–*C*). The *in silico* energy calculation also shows that the ΔG value for the sequential mismatch is less compared to TS1 DNA and perhaps due to this very little digestion occurs (Fig. 4*G*, Table S1*C*). Thus, with three or more sequential mutations, the activity of Cas9 is severely affected. Other sequential mutations from the 18th position onwards are also unable to digest. From the 19th position, there is a partial digestion. This shows that once triple sequential mutations move away from the Pam distal end, they inhibit the activity of Cas9. In the case of sequential mismatches, we observed two distinct scenarios. In one scenario, mutations resulted in significant RMSD values (Fig. 4*D*), indicating a notable decrease in stability (*e.g.*, 2017mm-TS1, 2015mm-TS1, 1816mm-TS1 with mean RMSD values exceeding 3 Å). Consequently, these mutants exhibited no detectable catalytic activity (<3% digestion). For instance, 2015mm-TS1 displayed a high mean RMSD value of 3.7 Å and a minimal digestion value of 3.97%. In the other scenario, these mutations did not induce substantial deviations in the duplex structure, leading to partial catalytic activity. For instance, 2018mm-TS1 had a mean RMSD of 2.8 Å with a digestion value of 86.1%. Upon superimposition, the initial conformation of the RNA-DNA duplex in 2015mm-TS1 displayed significant structural deviation from the average conformation, with an RMSD of 3.7 Å (Fig. 4*E*). In contrast, the RMSD for 2018mm-TS1 was quite low, at 2.8 Å (Fig. 4*F*) even though the number of mutations in 1816mm-TS1 and 2018mm-TS1 is the same.

### SpCas9–gRNA complex interaction with bi-sequentially mutated nucleotides in TS1

**Figure 5:**
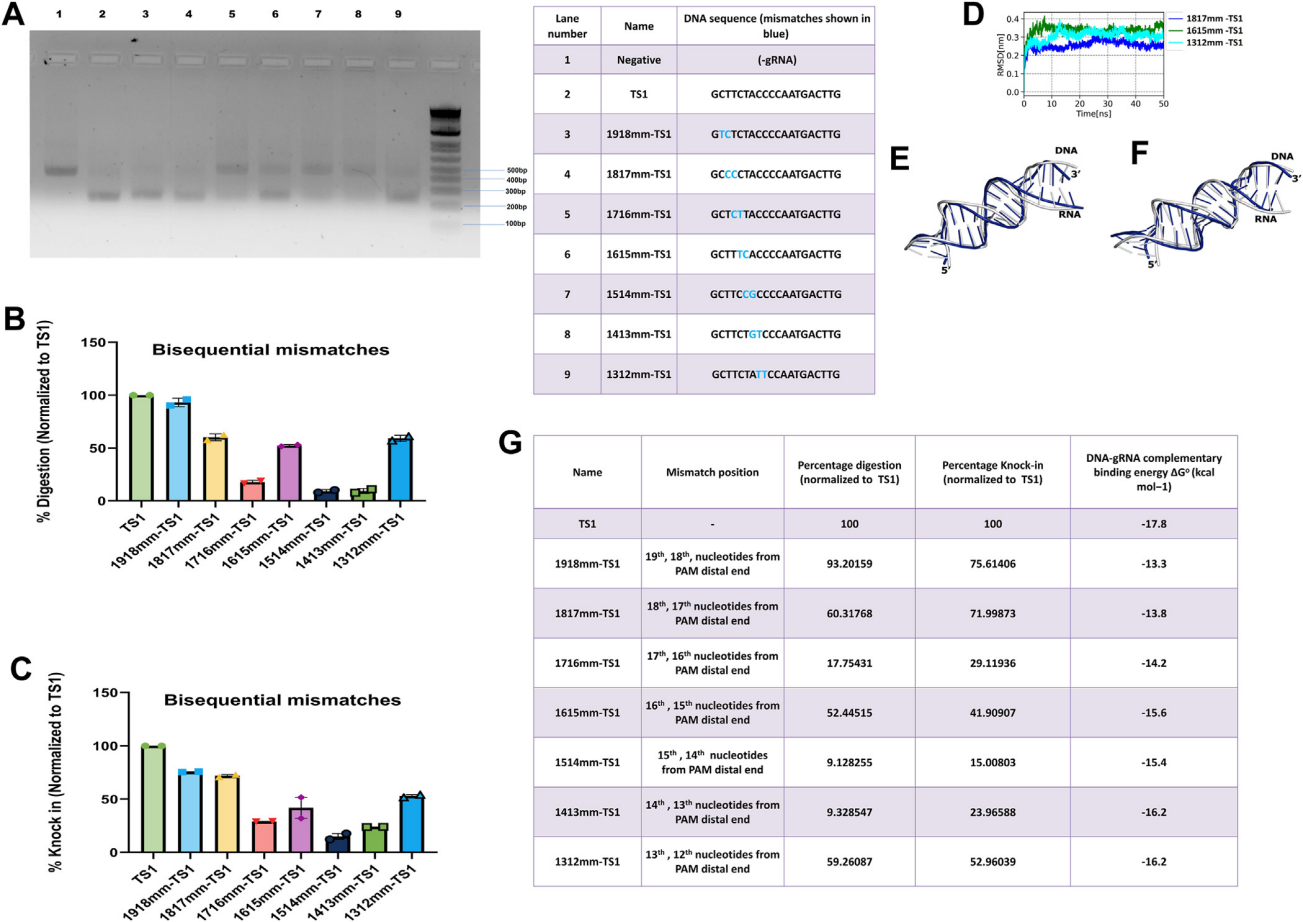
**Cas9 functional activity on encountering bi-sequential mismatches across various positions.***A*, DNA digestion of Cas9 with bi-sequential positional mismatches in the TS1 target DNA. *B*, graphical representation of the percentage digestion after 60 min. Data were normalized to TS1. *C*, graphical representation of the percentage of HDR knock in derived from cell-based reporter assay. Data was normalized to TS1. *D*, RMSD trajectory of the RNA-DNA duplex from bi-sequential mismatch mutants. Superimposition of initial and average conformation (computed from 40 ns–50 ns) of RNA-DNA duplex of (*E*) 1615mm-TS1 (RMSD 3.4 Å) and *F*) 1312mm-TS1 (RMSD 3.1 Å). *G*, tabular representation of nature of mismatch, normalized % of *in vitro* digestion, normalized %of knock in (cell-based reporter assay), and DNA-gRNA complementation derived energy.

The reports of single and sequential mutations are widely reported and are generally expected to be so. Our analysis of the physical effects on duplex strands upon mismatches is also in agreement with the Cas9 activity results. Cas9 behavior upon encountering sequential double nucleotide mismatches has been explored in this study as well. It was shown in the previous section that the mismatch 2019mm-TS1 located in the region of the 20th and 19th nucleotide still retains Cas9 activity. Energy analysis as well as the parameters of physical effect on the DNA strand was also checked. To further this study, we have designed several such mismatches (Table 2). Its effect has been studied from the 13th position to the 20th position localized in the PAM distal end. We report that Cas9 activity in positions 19 to 18 and positions 18 to 19 is retained (>60%). This is not surprising as even the triple sequential mutation from 20 to 18 base, at the PAM distal end, retained its activity. Mismatches in positions 17 to 14 are not well tolerated, as reported earlier (17). Double mismatch in the positions 16th and 15th showed partial activity while the rest showed no activity. Duplex parameters also showed significantly altered values when compared to the TS1. However, 1312mm-TS1 in position 13th and 12th from the PAM distal end showed full activity (Fig. 5, *A*–*C*). This was unexpected given the fact that this mutation is occurring in the seed region. Even the complementation-derived energy for the mismatched targets in this region is close to the TS1 (Fig. 5*G*, Table S1*B*) Given the current algorithm's practice of assigning importance to mismatch position, this particular mismatch may have been predicted as a non-tolerable mismatch. But as we can see from the Cas9 activity it is well tolerated. Herein lies the importance of studying the stability of the DNA-RNA duplex. Bi sequential mismatch mutants exhibited mean RMSD values ranging from 2.6 Å to 3.6 Å (Fig. 5*D*) and were classified as displaying low to partial catalytic activity (digestion values below 25% for 1716mm-TS1, 1514mm-TS1 and 1413mm-TS1 and 50% to 60% for 1817mm-TS1, 1615mm-TS1 and 1312mm-TS1). 1817mm-TS1, 1615mm-TS1and 1312mm-TS1 (Fig. 5, *E* and *F*) exhibited good overall structural alignment between their initial and average conformations, with RMSD values of 2.6 Å, 3.4 Å and 3.1 Å, respectively backing their activity range.

### SpCas9–gRNA complex interaction with staggered mutated nucleotides in TS1

**Figure 6:**
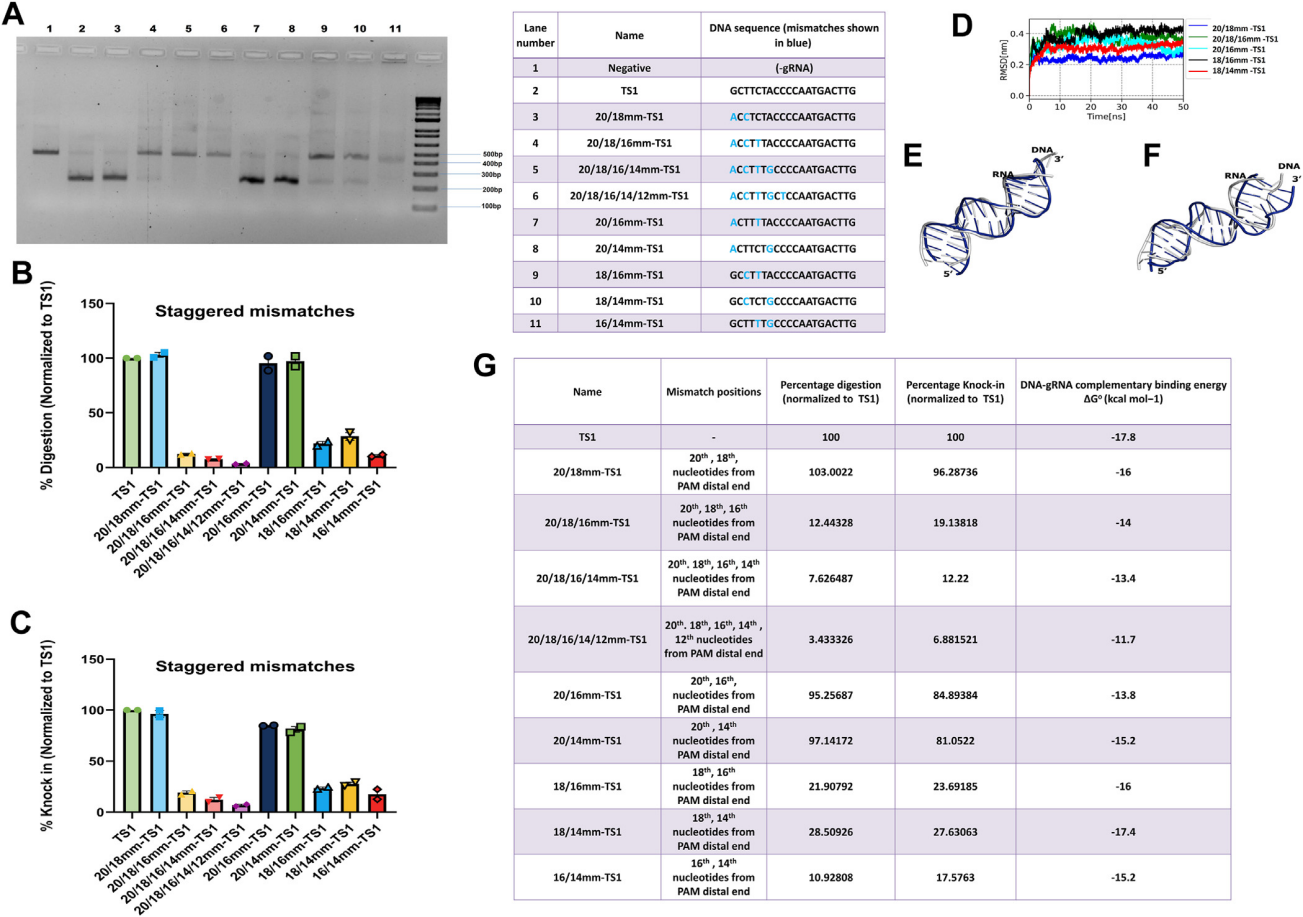
**Cas9 functional activity on encountering staggered mismatches across various positions.***A*, DNA digestion of Cas9 with staggered positional mismatches in the TS1 target DNA. *B*, graphical representation of the percentage digestion after 60 min. Data was normalized to TS1. *C*, graphical representation of the percentage of HDR knock in derived from cell-based reporter assay. Data was normalized to TS1. *D*, RMSD trajectory of the RNA-DNA duplex from staggered mismatch mutants. Superimposition of initial and average conformation (computed from 40 ns–50 ns) of RNA-DNA duplex of (*E*) 20/18mm-TS1 (RMSD 2.4 Å) and (*F*) 18/16mm-TS1 (RMSD 3.9 Å). *G*, tabular representation of nature of mismatch, normalized % of *in vitro* digestion, normalized %of knock in (cell-based reporter assay), and DNA-gRNA complementation derived energy.

The above mismatches reported in this study have been shown by other researchers as well (10, 11). we designed staggered mismatches *i.e.* mismatches that are separated from each other by various distances (Table 2). There are not many studies regarding these mismatches and they will serve as rigorous testing for the utility of duplex stability. The various mismatches have been listed in the table (Fig. 6*G*, Table S1*D*). Mismatches 20/18mm-TS1, 20/16mm-TS1 and 20/14mm-TS1 show Cas9 functional activity. It is interesting to note that the mismatches are being tolerated even with increasing encroachment to the interior. Mismatches 18/16mm-TS1, 18/14mm-TS1 are partially tolerated by Cas9 (∼30% digestion); however, 16/14mm-TS1 is not tolerated by Cas9 (∼10% digestion) (Fig. 6, *A*–*C*). This is hard to explain in terms of assigned positional significance. For instance, 20/18mm-TS1 exhibited a mean RMSD value of 2.4 Å and a digestion activity of ∼100%%, whereas 18/16mm-TS1 displayed a mean RMSD value of nearly 4 Å with ∼22% digestion activity (Fig. 6*D*). The structural alignment of these two mutants also supported these findings (Fig. 6, *E* and *F*).

**Figure 7:**
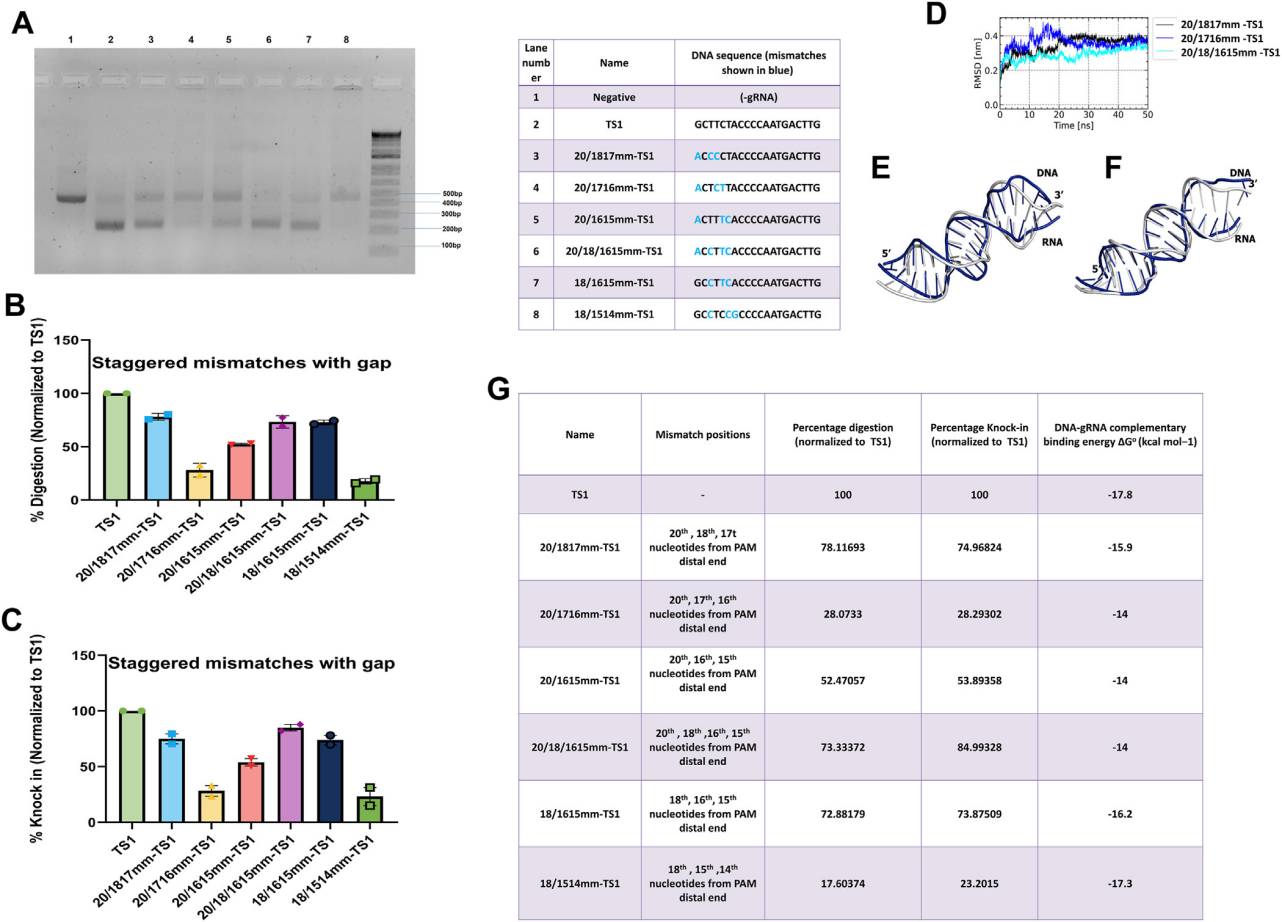
**Cas9 functional activity on encountering staggered mismatches containing single and double mismatches across various positions.***A*, DNA digestion of Cas9 with staggered positional mismatches containing single and double mismatches across various positions in the TS1 target DNA. *B*, graphical representation of the percentage digestion after 60 min. Data was normalized to TS1. *C*, graphical representation of the percentage of HDR knock in derived from cell-based reporter assay. Data were normalized toTS1. *D*, RMSD trajectory of the RNA-DNA duplex from staggered gap mismatch mutants. Superimposition of initial and average conformation (computed from 40 ns–50 ns) of RNA-DNA duplex of (*E*) 20/1716mm-TS1 (RMSD 3.6 Å) and *F*)20/18/1615mm-TS1 (RMSD 2.9 Å). *G*, tabular representation of nature of mismatch, normalized % of *in vitro* digestion, normalized %of knock in (cell-based reporter assay), and DNA-gRNA complementation derived energy.

We now wanted to see the effect when single and bi-sequential mismatches occur in the same target site. Mismatches 20/1817mm-TS1, 20/1716mm-TS1, and 20/18/1615mm-TS1 were studied for their Cas9 activity. We found that all the mismatched sites had activity. This is unexpected as it is in the nucleic acid binding domains region (17). 20/1716 mm -TS1 gave partial activity, while 20/1817mm-TS1 and 20/18/1615mm-TS1 gave almost full activity (Fig. 7, *A*–*C*). This was very interesting, especially the tolerance of 20/18/1615mm-TS1. Energy and previous positional reports are unable to explain this satisfactorily (Fig. 7*G*, Table S1*D*). Staggered gap mismatch mutants (20/1817mm-TS1 and 20/18/1615mm-TS1) fell into categories of moderate to high catalytic activity, with digestion values ranging from 60% to 80%. The mean RMSD value of 20/18/1615mm-TS1 was 2.9 Å which is lower than that of 20/1716mm-TS1 (3.6 Å) which explains the stability of the former and thus the higher activity (Fig. 7, *D*–*F*).

### SpCas9-gRNA complex interaction with mutated nucleotides in TS4 and TS5

In order to ascertain the generalizability of our findings beyond specific DNA sequences, we conducted additional experiments targeting two additional target sites, designated as TS4 and TS5, respectively. We systematically investigated a comprehensive array of mismatch configurations across these target sites, encompassing various types such as bi-sequential, sequential, staggered, and staggered with gap configurations, and also calculated the complementation-derived energy (Table S1, *G* and *H*). This rigorous examination, totaling 9 sets of DNA–RNA hybrids, revealed a consistent and analogous trend in both % Digestion and % Knock-in rates comparable to those observed at the TS1 locus (Supplementary S2 and S3).

Subsequently, we employed Molecular Dynamics (MD) simulations to probe the structural dynamics of the DNA–RNA duplexes formed at select mismatched target sites. Notably, we observed a compelling association between the average RMSD of the DNA–RNA duplexes and the corresponding % Digestion rates. For instance, TS4 exhibited complete digestion (% Digestion: 100%) alongside an RMSD value of 2.1 Å, whereas the 18/1514mm-TS4 variant displayed minimal digestion (10%) concomitant with a higher RMSD of 3.8 Å. Remarkably, the 20/18/1615mm-TS4 variant exhibited heightened activity (∼80%) alongside a lower RMSD value of 2.9 Å.

## Discussion

CRISPR-Cas9 has demonstrated remarkable potential in precision genome editing, garnering its inaugural FDA approval for therapeutic intervention in 2023 (20). However, its widespread therapeutic application remains a distant goal, due to the significant challenge posed by its unprecedented off-target effects. While the generation of "knock-out cells" using CRISPR-Cas9 technology has become routine practice in modern cellular biology, it is mainly restricted to *in vitro* models. Applications of CRISPR-Cas9 have been done in the mice model; but, its efficacy is low and is prone to complications (21, 22). However in the mice model, this problem was addressed by Easi-CRISPR, a method developed by Quadros M. *et al.* (23), but still, non-specific cleavage of genomic DNA can cause cellular toxicity (24). Their subsequent repair often results in deletions, inversions, and translocations (25, 26, 27, 28). These mutations may cause modulation of gene expression of non-targeted essential genes in the human cell.

Many attempts have been made to modify the Cas9-gRNA complex to reduce the undesired off-target activity. However, such modifications have a sequence-dependent effect on SpCas9 activity (14, 18, 19, 29, 30). Moreover, the total GC content of the target DNA-gRNA hybrid had a significant effect on the SpCas9 activity. It was found from our experiments that for target DNAs having abnormally high or low GC content (<20% and >80%) reducing the length of the gRNA probe even below 17 nucleotides retained activity. In modified Cas9-gRNA complexes the off-target effect varies with the target DNA sequences but uniform effectiveness is desired for therapeutic application. For this, we need to be able to predict probable off-target possibilities reliably. Mismatches at the seed region of the Target DNA are not tolerated, whereas from our data it was found that mutations up to certain limits are tolerated at the PAM distal end and thus have a significant implication on the off-target effect of SpCas9. There are many recent off-target probability prediction algorithms including CCTop, CFD, CROP-IT, DeepCRISPR, Elevation, CRISPR-OFF, CRISPR, iGWOS, uCRISPR, and CRISPRater (31, 32, 33, 34, 35, 36, 37, 38, 39). Out of these DeepCRISPR, iGWOS and Elevation are based on machine learning, while the rest are based on energetic analysis and experimental rule-driven. However, none of these algorithms take into account the role of DNA-gRNA structural stability that arises due to mismatched target sites. In this present work, we attempted to find the role of DNA: RNA complementation derived energy and structural stability on their functional activity. We employed various PAM distal mutations in various DNA sequences and evaluated the roles of these mismatches on SpCas9 functionality using *in vitro* cleavage assay as well as cell-based reporter assay. We got very good correlations among them. *In vitro* assays have the benefit of simplification of complex phenomena and allowing avoidance of confounding factors, but they do not necessarily reflect the actual processes operating inside a cell. However, the cell-based experiment data also supported and endorsed *in vitro* data.

**Figure 8:**
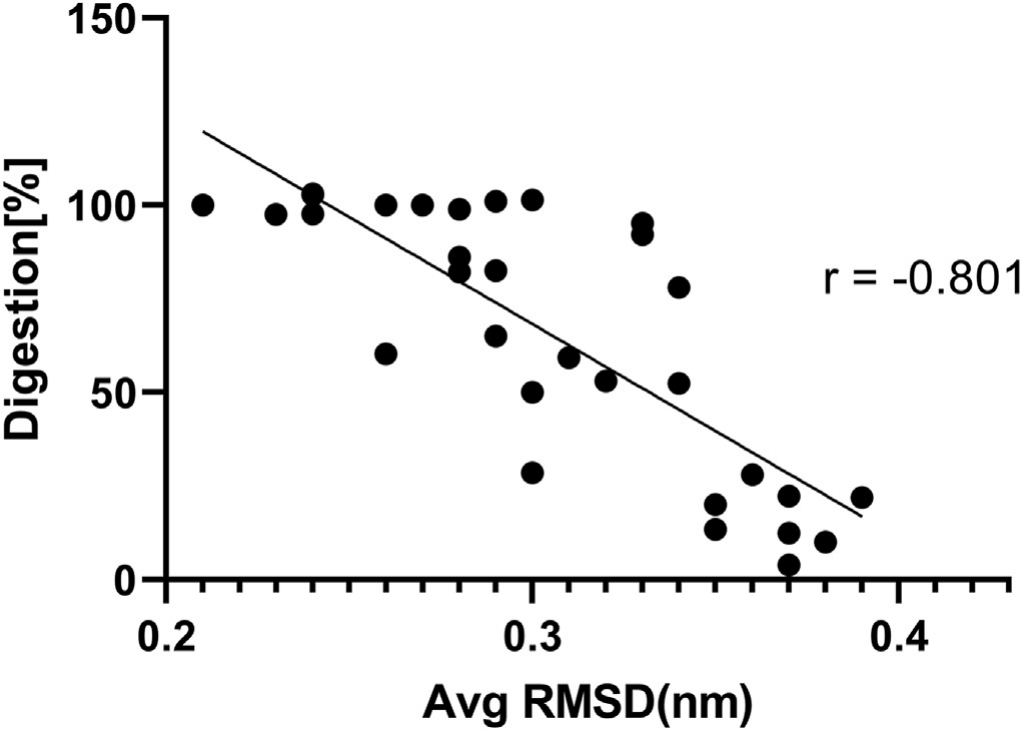
**Linear correlation plot that showcases the connection between the mean Root Mean Square Deviations (RMSDs) of the DNA-RNA duplex and the corresponding digestion percentages (catalytic activity).** The data shows strong negative correlation between %Digestion and average RMSD with a Pearson correlation coefficient (r) of −0.801.

Fu, Y., *et al.*, have shown that shorter probe length leads to less off-target effect as less energy is available. They have shown that 16 nucleotides result in no activity in concurrence with our result (17). A single base loss (*via* mismatch or deletion) is poorly tolerated when the probe length is 17 nucleotides (40). On the other hand, when the probe length was 20, single nucleotide mismatch tolerance occurred in the tail region across all positions. However, when the GC content was altered Cas9 activity altered despite shortening the length of the gRNA probe, by previously reported results (14). Single nucleotide mismatch has also been previously reported to be better tolerated (10, 11, 41). Many CRISPR-Cas9 off-target evaluators additionally focused on the position of the mismatch and the closer the mismatch is towards the PAM proximal end, the lesser the probability of off-target effect (33, 34, 35). We observed single mismatches at the PAM distal end do not have any significant difference in activity among them. Thus, there is no positional significance or weight attached to single mismatches occurring in this region. We also observed that bi-sequential mismatches were tolerated when they were present at the PAM distal end positions but the same was poorly tolerated when the mismatches were present in the 18–15th position from the PAM distal end. However, multiple sequential mismatches are poorly tolerated and upon an increase in mismatch to more than three nucleotides, there is a complete loss of activity which is at par with the previous reports by Ricci, C.G *et al.* and Hsu, P *et al.* (11, 42). It was observed from the data that 3 consecutive mismatches are poorly tolerated when the position of the mismatch is in the region of 18-15 nucleotides upstream of the PAM; however, a similar number of mismatches in the 5′end *i.e.* in the region of 20-18th position upstream of the PAM was tolerated and Cas9 remained functional. To highlight the importance of DNA-RNA duplex stability or instability in Cas9 activity, we designed a wide variety of mismatches that were staggered (gaps between mismatches). We found that some of these mismatches to be tolerated by Cas9 which could not be explained based on energy or positional effect. We have used MD simulation to characterize how specific mutations in the DNA sequence impact the function of CRISPR-Cas9. The choice of the 5Y36 structure as a model was significant because of its cleavage-compatible conformation. One of the key takeaways was the effect of these mutations on catalytic activity. We have computed the RMSD of RNA-DNA duplex from the trajectory as a measure of duplex stability. When single mismatch mutations were introduced, they didn't disrupt the structure of the RNA-DNA duplex significantly as indicated by low RMSD values. This was at par with our SpCas9 digestion assay findings. This meant that the system remained stable, and the SpCas9 enzyme could carry out its cleaving function efficiently, with digestion rates exceeding 85%. In essence, minor errors in the DNA sequence were tolerated quite well. For instance, single mismatches irrespective of their position were tolerated. However, things got more interesting when sequential mismatch mutations were examined. In some cases, these mutations led to noticeable structural deviations in the duplex, reducing the stability. As a result, the mutants where 3 or more mismatches are present at a stretch except at the 5′ terminals displayed little to no catalytic activity. For instance, 2015mm-TS1 (6 mismatched bases) had a high RMSD value and a minimal digestion rate, similarly, 1816mm-TS1 and 1715mm-TS4 showed low activity with high RMSD. The staggered mismatch mutants revealed another intriguing aspect. They fell into two categories: highly stable duplexes with high catalytic activity and considerably less stable duplexes with low activity. 20/16mm-TS1 and 20/16mm-TS4, for instance, both had low RMSD and a high digestion rate, while 18/16mm-TS1 had a high RMSD and no digestion activity. Staggered gap mismatch mutants also showed varying catalytic activities, ranging from low activity to high activity. For example, both 20/18/1615mm-TS1 and 20/18/1615mm-TS4 had multiple mismatches but interestingly they showed high catalytic activities and the average RMSD was also found to be low. To test the generalizability of our hypothesis, we have tested 2 sets of already published target sites (13), TS1 and TS4, and their mutations and we have established a linear correlation between the average RMSD values and the digestion percentage (dataset given in Supplementary S4). The calculated Pearson correlation coefficient (r) was −0.801 (Fig. 8). This finding suggests that an elevated RMSD value signifies greater inherent conformational instability within the RNA-DNA duplex structure, leading to a reduction in catalytic activity. Conversely, a lower RMSD value is associated with stable conformation and higher catalytic activity. Higher stability leads to the precise positioning of the scissile P atom in the catalytic site of the HNH domain and *vice versa*.

Overall, a significant correlation was established between the RMSD values and catalytic activity. This indicates that higher RMSD values, reflecting structural instability, were associated with reduced catalytic activity, while lower RMSD values indicated stability and higher activity. Importantly, the study also assessed the conformational dynamics of the RuvC and HNH domains. It found minimal conformational deviations, suggesting that the mutations in the DNA sequence did not significantly alter the dynamics of these catalytic domains. We observed that staggered mismatches having 3 mismatches but with at least 2 nucleotide gaps between the mismatches were tolerated pointing to the fact that the effect of mismatches is limited to the mutated base only. Our work showed that along with the energetics requirement and mismatch position, DNA-RNA duplex instability arising out of mismatches should also be taken into account for designing more specific gRNAs and thus reducing the off-target SpCas9 activity.

## Experimental procedures

### Cloning of target sites

All the target sites were cloned in our in-house designed reporter plasmid that is also used for cell-based assays (detailed description is given in section 4.7) Target site (containing various mismatches- Table 2) oligonucleotides and their complimentary oligonucleotides were annealed by heating them at 95^o^C and gradually reducing the temperature by 4 °C in a touchdown manner where each cycle is of 4 min. The final temperature is 4 °C at which the annealed product is obtained and phosphorylated. The reporter assay plasmid was sequentially double digested with EcoRI and Xho1 at 37 °C for 2 h and dephosphorylated. The digested gel product was purified and ligated with 1:100 dilution of the annealed product using T4 DNA ligase. The ligation reaction was performed overnight at 16 °C. The ligated product was then transformed into XL1B cells with ampicillin selection. The obtained colonies were screened by sequencing the plasmids isolated from them. After successful cloning of the target sites in reporter assay plasmid, the target DNA for *in vitro* experiments was obtained using PCR amplification. The length of the amplicon in each case was 450 bp with the target at the middle portion. Therefore, after SpCas9 mediated DNA double-strand break, the cleaved DNAs formed were of 225 bp approx. This can be observed in all the agarose gel images in Figure 2, Figure 3, Figure 4, Figure 5, Figure 6, Figure 7, Figure 8. However, in panel A of Figure 2 we used different primers to depict the individual DNA fragments after SpCas9 digestion.

### Cloning of probe region in gRNA

Probe regions and their complementary primers (Table 3) were annealed by heating them at 95 °C and gradually reducing the temperature by 4 °C in a touchdown manner where each cycle is of 4 min. The final temperature is 4 °C at which the annealed product is obtained and phosphorylated. pT7gRNA plasmid (a gift from Wenbiao Chen having Addgne ref #46759) was digested with BsmBI at 55 °C for 2 h and dephosphorylated. The digested gel product was purified and ligated with 1:100 dilution of the annealed product using T4 DNA ligase. The ligation reaction was performed overnight at 16 °C. The ligated product was then transformed into XL1B cells with ampicillin selection. The obtained colonies were screened by sequencing the plasmids isolated from them.

### Synthesis and purification of gRNAs

The cloned plasmids were digested with BamHI and the digested product was purified by gel purification using a QIAquick Gel Extraction Kit (Qiagen, Cat No-28706). The linearized vector was then used in an IVT reaction using T7 RNA polymerase to produce gRNA. The produced gRNA was purified using a Qiagen RNA purification kit (RNeasy Mini Kit, Qiagen, cat no-74104) after DNase I digestion. The purified gRNA was checked for integrity on an 8% Urea Page.

### Expression of SpCas9

pET-28b-Cas9-His (a gift from Alex Schier (Addgene plasmid # 47327))was obtained from addgene) and it was transformed in BL21DE3 Rossetta pLysS cells with kanamycin resistance. The transformed cells were inoculated in LB overnight at 37 °C. The overnight cells were again inoculated in a 1-L culture. Upon reaching a growth O.D. of 0.6, the cells were induced with IPTG at a final concentration of 1 mM. The induced cells were grown overnight at 17 °C and after checking for induction the cells were pelleted and proceeded for the purification step.

### Purification of SpCas9

The pelleted induced cells were resuspended with lysis buffer (50 m Tris pH 8.0, 150 mM NaCl, 10 mM MgCl2, 0.1% NP 40) and incubated in ice for 30 min. Post-incubation the cells were sonicated and centrifuged at 16,000*g* for 30 min at 4 °C. The supernatant was collected and charged on a Ni-NTA column at 4 °C. The column was washed with various concentrations of Imidazole. The proteins were then eluted at 300 mM imidazole and the eluted protein was dialyzed in dialysis buffer (50 m Tris pH 8.0, 150 mM NaCl, 10 mM MgCl2, 0.1% NP 40, and 20% glycerol). Dialysis was performed at 4^o^C overnight.

### SpCas9 functional assay

We first synthesized different-sized gRNA by *in vitro* transcription. The RNA was then purified and checked for integrity on a urea-PAGE. The three individual components *i.e.* the SpCas9 protein, gRNA, and target DNA were reconstituted and incubated for 60 min at 37 °C. After incubation was done the reaction was stopped by incubating the mixture at 65 °C for 15 min. Post this, the samples were first incubated with RNase A at 37 °C for 15 min. After this, they were incubated with Proteinase K at 55 °C for another 15 min. Finally, the samples were then run on a 2% Agarose gel. Each experiment was replicated twice independently.

### Designing of cell-based reporter assay system

A cell-based reporter assay plasmid was constructed such that m-cherry and the first 474 nucleotides of GFP are presented in the frame under CMV promoter. Then it is followed by a stop codon and the rest of the nucleotide sequence of the GFP is present that is out of frame. EcoRI and XhoI digestion sites are present flanking the stop codon and between these two restriction sites, the desired target sites are cloned. The reporter assay plasmid is designed such that mCherry is always expressed upon transfection but GFP is not expressed as such. Upon co-transfection of the reporter assay plasmid containing the target sites, the CRISPR-Cas9-gRNA plasmid (pX330-U6-Chimeric_BB-CBh-hSpCas9) and Homology arm (as a purified PCR product) the entire sequence of GFP comes in the frame (due homologous recombination after Cas9 mediated DNA double-strand break) and only then GFP is expressed. The homology arm was amplified from pX458 plasmid (pSpCas9(BB)-2A-GFP (pX458) was a gift from Feng Zhang -Addgene plasmid # 48138) using a set of primers having approximately 200 bp right side and left side homology respectively spanning the cut site. The corresponding gRNAs for the target sites TS1, TS4 and TS5 were cloned in the pX330-U6-Chimeric_BB-CBh-hSpCas9 plasmid (a gift from Feng Zhang Addgene plasmid # 42230) according to the protocol described by Cong *et al.* (8) Thus, cells which express both mCherry and GFP (dual expression) serve as positive control which indicate successful CRISPR-Cas9 mediated homologous knock-in (Fig. 1*A*).

### Transfection and flow cytometry analysis

HEK 293 cells were maintained in DMEM High Glucose supplemented with 10% FBS and 1% Antibiotic-Antimycotics. The aforementioned reporter assay plasmid containing the various target sites, Homologous arm as Purified PCR product, and plasmid encoding gRNA and Cas9 were co-transfected using Lipofectamine 2000 (Invitrogen). The amount of the various DNAs was standardized previously and was added in corresponding wells in a 24-well tissue culture plate. Following the addition of the DNAs, the lipofectamine solution (1:100) in opti-mem media (Gibco) was added to each well and at last, 10^5^ HEK 293 cells were added to each well. 4 h post transfection, the entire opti-mem media was discarded and fresh DMEM High glucose media was added. The transfected cells were maintained for 48 h and then Flowcytometric analysis of homologous Knock-in was performed using FACS (LSR Fortessa, BD bioscience). The cells that were transfected with the reporter assay system expressed mcherry that was confirmed by the population observed in quadrant Q1 (Fig. 1*B*). The sub-population of transfected cells in which homologous recombination occurred due to SpCas9 mediated DNA double-strand break and repair expressed GFP. this was evident from the dots observed in quadrant Q2 (Fig. 1*C*). The frequency of Knock-in was calculated using the formula mentioned in Figure 1*D*. In each instance the data from 10,000 cells are plotted in the dot plot from which the % knock-in was calculated and enlisted in panel G of Figure 3, Figure 4, Figure 5, Figure 6, Figure 7. Each experiment was repeated twice or thrice for statistical significance.

### DNA pull-down with SpCas9-gRNA complex

The three individual components *i.e.* the SpCas9 protein, gRNA, and target DNA were reconstituted and incubated for 30 min. After that Ni-NTA beads, equilibrated in reaction buffer, was added in equal amount to all the reaction. This was further incubated for another 30 min at room temperature. Post incubation, the beads were washed twice with reaction buffer. 15 μl of reaction buffer was added to all the beads and heated to 65 °C for 15 min. The samples were first incubated with RNase A at 37 °C for 15 min. After this, they were incubated with Proteinase K at 55 °C for another 15 min. The samples were then run on an 8% native page and their intensity was plotted. For the bead control set, gRNA without probe was used. The reactions were done in duplicate.

### *In silico* energy analysis

We have calculated delta G (ΔG°) or Gibbs free energy for the guide RNA (gRNA) by utilizing the nearest-neighbor (NN) model which predicts DNA/RNA thermodynamics by utilizing energy values for different base pair motifs. We have used the link Nearest Neighbor Calculator for Nucleic Acids (konan-fiber.jp) for these calculations (43, 44, 45, 46).

### Structure preparation and molecular dynamics simulation

SpCas9-sgRNA-DNA ternary complex structure was taken from PDB (pdb id: 5Y36) and any missing atoms were added using CHARMM-GUI server (47). We have replaced the original guideRNA and target DNA sequence of the pdb structure with our own guideRNAs and target DNA sequences using w3DNA 2.0 webserver (48). Following the generation of the server, several mismatched nucleotides in the target DNA strands of TS1, TS4, and 2 sets of target DNA from already published paper (13) were introduced using the same server resulting in 32 independent DNA-RNA hybrid systems with different nucleotide sequences (Fig. 1*E*). We have depicted the RNA-DNA duplex model including the cut site phosphate atom in Figure 1*F*. Molecular dynamics simulation was conducted for total 32 systems. GROMACS 2022 (49) engine was used for the all the simulations. AMBER99-IILDN (50) force field was used in this study and the temperature was set to 300 K. All the systems were placed in a dodecahedron box with box edge of 12 Å and 8 Å distance was set between protein atoms and box edge. All the systems were solvated using TIP3 water models and the solvated system were neutralized by adding sufficient numbers of Na+ and Cl-counter ions. All the systems were energy minimized using steepest descent algorithm with 50,000 steps. The minimization step is continued until the maximum force on the atoms is less than 1000 kJ/mol/nm. Then we performed 100 ps of NVT equilibration step in order to equilibrate the system at 300 K temperature using the Langevin thermostat (51) and subsequently 100 ps of NPT equilibration in order to equilibrate it at 1 atm pressure using Berendsen barostat (52). During the NVT and NPT equilibration, all the heavy atoms were restrained with a positional constraint of 1000 kcal/mol Å-2. Production run for each system was conducted for 50 ns in the NPT ensemble with no restraint resulting in a total 1000 ns of simulation. To handle long-range Coulomb interactions, the particle mesh Ewald summation method (PME) (53) was employed by setting the mesh spacing to 1.0 Å. All the analyses were done using the inbuild gromacs command line tool and plotting was done using Python matplotlib module.

### Statistical analysis

All data furnished in this manuscript are mean value ± SEM of multiple independent experiments and for linear correlation among 2 parameters linear correlation was performed where *p* value (two-tailed) was <0.0001. All statistical calculations were performed using Graphpad Prism (version 9.5.1).

## Data availability

Authors agree to provide materials, used in our study, promptly available to others upon request.

## Supporting information

This article contains supporting information.

## Conflict of interest

The authors declare that they have no known competing financial interests or personal relationships that could have appeared to influence the work reported in this paper.

## Figures and Tables

**supplementary s2.jpg:**
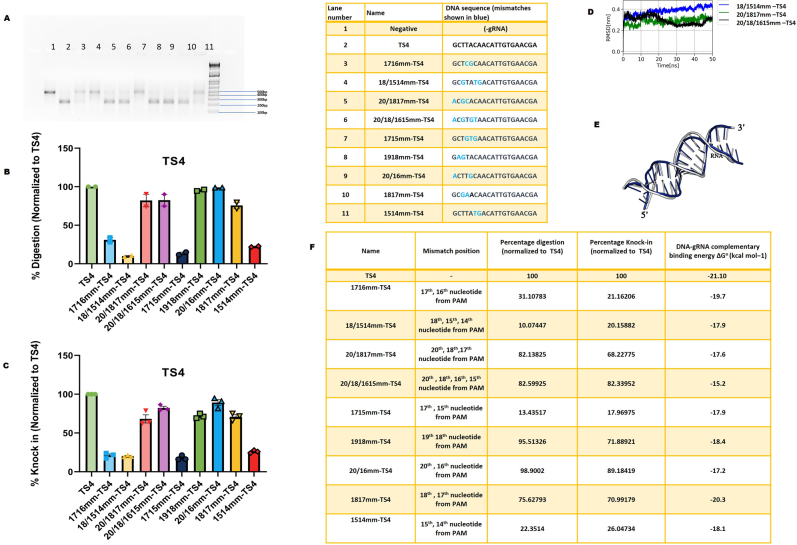


**supplementary s3.jpg:**
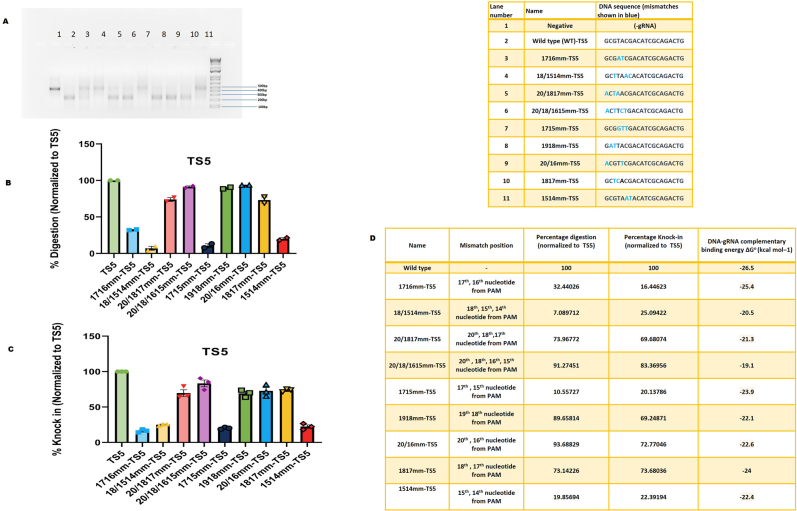


## Supporting information

S1.docx

S2_S3 legends.docx
