# Supplementary material for: A mechanistic study on the tolerance of PAM distal end mismatch by SpCas9
Source: J Biol Chem. 2024 Jun 3;300(7):107439. doi: 10.1016/j.jbc.2024.107439 (PMC11267045; doi:10.1016/j.jbc.2024.107439)
Supplement: S1.docx [file mmc1.docx]

**SUPPLEMENTARY TABLE- DNA-RNA complimentary binding energy.**

**Table A-single mismatches**

| **NAME** | **SEQUENCE (5’ > 3’)** | **ΔG^o^ (kcal mol−1)** |
| --- | --- | --- |
| **TS1** | **GCTTCTACCCCAATGACTTG** | **-17.8** |
| **20MM** | **ACTTCTACCCCAATGACTTG** | **-15.8** |
| **19MM** | **GTTTCTACCCCAATGACTTG** | **-16.8** |
| **18MM** | **GCCTCTACCCCAATGACTTG** | **-18** |
| **17MM** | **GCTCCTACCCCAATGACTTG** | **-18** |
| **16MM** | **GCTTTTACCCCAATGACTTG** | **-15.8** |
| **15MM** | **GCTTCCACCCCAATGACTTG** | **-18.1** |
| **14MM** | **GCTTCTGCCCCAATGACTTG** | **-17.2** |
| **13MM** | **GCTTCTATCCCAATGACTTG** | **-16.1** |

**Table B -Bi-Sequential Mismatch**

| **NAME** | **SEQUENCE (5’ > 3’)** | **ΔG^o^ (kcal mol−1)** |
| --- | --- | --- |
| **TS1** | **GCTTCTACCCCAATGACTTG** | **-17.8** |
| **2019MM** | **ATTTCTACCCCAATGACTTG** | **-15.4** |
| **1918MM** | **GTCTCTACCCCAATGACTTG** | **-17** |
| **1817MM** | **GCCCCTACCCCAATGACTTG** | **-17.9** |
| **1716MM** | **GCTCTTACCCCAATGACTTG** | **-16** |
| **1615MM** | **GCTTTCACCCCAATGACTTG** | **-16** |
| **1514MM** | **GCTTCCGCCCCAATGACTTG** | **-17.1** |
| **1413MM** | **GCTTCTGTCCCAATGACTTG** | **-15.5** |
| **1312MM** | **GCTTCTATTCCAATGACTTG** | **-14.4** |

**Table C- Sequential Mismatches and triple mismatches**

| **NAME** | **SEQUENCE (5’ > 3’)** | **ΔG^o^ (kcal mol−1)** |
| --- | --- | --- |
| **TS1** | **GCTTCTACCCCAATGACTTG** | **-17.8** |
| **2018MM** | **ATCTCTACCCCAATGACTTG** | **-15.6** |
| **2017MM** | **ATCCCTACCCCAATGACTTG** | **-14.2** |
| **2016MM** | **ATCCTTACCCCAATGACTTG** | **-13.8** |
| **2015MM** | **ATCCTCACCCCAATGACTTG** | **-13.3** |
| **1917MM** | **GTCCCTACCCCAATGACTTG** | **-16.2** |
| **1816MM** | **GCCCTTACCCCAATGACTTG** | **-16.2** |
| **1715MM** | **GCTCTCACCCCAATGACTTG** | **-16.2** |

**Table D- Staggered Mismatches**

| **NAME** | **SEQUENCE (5’ > 3’)** | **ΔG^o^ (kcal mol−1)** |
| --- | --- | --- |
| **TS1** | **GCTTCTACCCCAATGACTTG** | **-17.8** |
| **20/18MM** | **ACCTCTACCCCAATGACTTG** | **-16** |
| **20/18/16MM** | **ACCTTTACCCCAATGACTTG** | **-14** |
| **20/18/16/14MM** | **ACCTTTGCCCCAATGACTTG** | **-13.4** |
| **20/18/16/14/12MM** | **ACCTTTGCTCCAATGACTTG** | **-11.7** |
| **20/16MM** | **ACTTTTACCCCAATGACTTG** | **-13.8** |
| **20/14MM** | **ACTTCTGCCCCAATGACTTG** | **-15.2** |
| **18/16MM** | **GCCTTTACCCCAATGACTTG** | **-16** |
| **18/14MM** | **GCCTCTGCCCCAATGACTTG** | **-17.4** |
| **16/14MM** | **GCTTTTGCCCCAATGACTTG** | **-15.2** |
| **20/1817MM** | **ACCCCTACCCCAATGACTTG** | **-15.9** |
| **20/1716MM** | **ACTCTTACCCCAATGACTTG** | **-14** |
| **20/1615MM** | **ACTTTCACCCCAATGACTTG** | **-14** |
| **20/18/1615MM** | **ACCTTCACCCCAATGACTTG** | **-14.2** |
| **18/1615MM** | **GCCTTCACCCCAATGACTTG** | **-16.2** |
| **18/1514MM** | **GCCTCCGCCCCAATGACTTG** | **-17.3** |

**Table E- TS2**

| **NAME** | **SEQUENCE (5’ > 3’)** | **ΔG^o^ (kcal mol−1)** |
| --- | --- | --- |
| **TS2** | **CGCGGCGGCGTCAGGCACCT** | **-28.6** |

**Table F- TS3**

| **NAME** | **SEQUENCE (5’ > 3’)** | **ΔG^o^ (kcal mol−1)** |
| --- | --- | --- |
| **TS3** | **TATAATAATACTGAATGTTC** | **-12.9** |

**Table G- TS4**

| **NAME** | **SEQUENCE (5’ > 3’)** | **ΔG^o^ (kcal mol−1)** |
| --- | --- | --- |
| **TS4** | **GCTTACAACATTGTGAACGA** | **-21.1** |
| **1716mm-TS4** | **GCTCGCAACATTGTGAACGA** | **-19.7** |
| **18/1514mm-TS4** | **GCGTATGACATTGTGAACGA** | **-17.9** |
| **20/1817mm-TS4** | **ACGCACAACATTGTGAACGA** | **-17.6** |
| **20/18/1615mm-TS4** | **ACGTGTAACATTGTGAACGA** | **-15.2** |
| **1715mm-TS4** | **GCTGTGAACATTGTGAACGA** | **-17.9** |
| **1918mm-TS4** | **GAGTACAACATTGTGAACGA** | **-18.4** |
| **20/16mm-TS4** | **ACTTGCAACATTGTGAACGA** | **-17.2** |
| **1817mm-TS4** | **GCGAACAACATTGTGAACGA** | **-20.3** |
| **1514mm-TS4** | **GCTTATGACATTGTGAACGA** | **-18.1** |

**Table H- TS5**

| **NAME** | **SEQUENCE (5’ > 3’)** | **ΔG^o^ (kcal mol−1)** |
| --- | --- | --- |
| **TS5** | **GCGTACGACATCGCAGACTG** | **-26.5** |
| **1716mm-TS5** | **GCGATCGACATCGCAGACTG** | **-25.4** |
| **18/1514mm-TS5** | **GCTTAACACATCGCAGACTG** | **-20.5** |
| **20/1817mm-TS5** | **ACTAACGACATCGCAGACTG** | **-21.3** |
| **20/18/1615mm-TS5** | **ACTTCTGACATCGCAGACTG** | **-19.1** |
| **1715mm-TS5** | **GCGGTTGACATCGCAGACTG** | **-23.9** |
| **1918mm-TS5** | **GATTACGACATCGCAGACTG** | **-22.1** |
| **20/16mm-TS5** | **ACGTTCGACATCGCAGACTG** | **-22.6** |
| **1817mm-TS5** | **GCTCACGACATCGCAGACTG** | **-24** |
| **1514mm-TS5** | **GCGTAATACATCGCAGACTG** | **-22.4** |
