# Supplementary material for: A mechanistic study on the tolerance of PAM distal end mismatch by SpCas9
Source: J Biol Chem. 2024 Jun 3;300(7):107439. doi: 10.1016/j.jbc.2024.107439 (PMC11267045; doi:10.1016/j.jbc.2024.107439)
Supplement: S2_S3 legends.docx [file mmc2.docx]

*Supplementary S2 – Cas9 functional activity on encountering mismatches across various positions in TS4*

*A) Cas9 digestion activity of TS4 and its mutated versions*

*B) Graphical representation of the percentage digestion after 60 mins. Data was normalized to TS4*

*C) Graphical representation of the percentage of HDR knock in derived from cell-based reporter assay. Data was normalized to TS4*

*D) RMSD trajectory of the RNA-DNA duplex from TS4 set.*

*E) Superimposition of initial and average conformation (computed from 40ns-50ns) of RNA-DNA duplex of 20/1817mm-TS4 (2.8 Å)*

*F) Tabular representation of nature of mismatch, normalized % of in vitro digestion, normalized %of knock in (cell-based reporter assay), and DNA-gRNA complementation derived energy.*

*Supplementary S3 – Cas9 functional activity on encountering mismatches across various positions*

*A) Cas9 digestion activity of various mismatched DNA is similar to their corresponding mismatched DNAs across other target sites (TS1 and TS4)*

*B) Graphical representation of the percentage digestion after 60 mins. Data was normalized TS5*

*C) Graphical representation of the percentage of HDR knock in derived from cell-based reporter assay. Data was normalized to TS5*

*D) Tabular representation of nature of mismatch, normalized % of in vitro digestion, normalized %of knock in (cell-based reporter assay), and DNA-gRNA complementation derived energy.*
